# Multiclass Sparse Bayesian Regression for fMRI-Based Prediction

**DOI:** 10.1155/2011/350838

**Published:** 2011-06-23

**Authors:** Vincent Michel, Evelyn Eger, Christine Keribin, Bertrand Thirion

**Affiliations:** ^1^PARIETAL Team, INRIA Saclay-*Î*le-de-France, 91191 Saclay, France; ^2^Laboratoire de Mathématiques, Université Paris-Sud 11, 91400 Orsay, France; ^3^CEA, DSV, I2BM, NeuroSpin, 91191 Gif-sur-Yvette, France; ^4^CEA, DSV, I2BM, INSERM U562, 91191 Gif-sur-Yvette, France; ^5^SELECT Team, INRIA Saclay-*Î*le-de-France, 91400, France

## Abstract

*Inverse inference* has recently become a popular approach for analyzing neuroimaging data, by quantifying the amount of information contained in brain images on perceptual, cognitive, and behavioral parameters. As it outlines brain regions that convey information for an accurate prediction of the parameter of interest, it allows to understand how the corresponding information is encoded in the brain. However, it relies on a prediction function that is plagued by the curse of dimensionality, as there are far more features (voxels) than samples (images), and dimension reduction is thus a mandatory step. We introduce in this paper a new model, called *Multiclass Sparse Bayesian Regression* (*MCBR*), that, unlike classical alternatives, automatically adapts the amount of regularization to the available data. MCBR consists in grouping features into several classes and then regularizing each class differently in order to apply an adaptive and efficient regularization. We detail these framework and validate our algorithm on simulated and real neuroimaging data sets, showing that it performs better than reference methods while yielding interpretable clusters of features.

## 1. Introduction

In the context of neuroimaging, machine learning approaches have been used so far to address diagnostic problems, where patients were classified into different groups based on anatomical or functional data. By contrast, in cognitive studies, the standard framework for functional or anatomical brain mapping was based on mass univariate inference procedures [1]. Recently, a new way of analyzing functional neuroimaging data has emerged [2, 3], and it consists in assessing how well behavioral information or cognitive states can be predicted from brain activation images such as those obtained with functional magnetic resonance imaging (fMRI). This approach opens new ways for understanding the mental representation of various perceptual and cognitive parameters, which can be regarded as the study of the corresponding *neural code*, albeit at a relatively low spatial resolution. The accuracy of the prediction of the behavioral or cognitive target variable, as well as the spatial layout of predictive regions, can provide valuable information about functional brain organization; in short, it helps to *decode* the brain system [4].

Many different pattern recognition and machine leaning methods have been used to extract information from brain images and compare it to the corresponding target. Among them, *Linear Discriminant Analysis (LDA)* [3, 5], *Support Vector Machine (SVM)* [6–9], or regularized prediction [10, 11] has been particularly used. The major bottleneck in this kind of analytical framework is that there are far more features than samples, so that the problem is plagued by the curse of dimensionality, leading to overfitting. Dimension reduction can be used to extract relevant information from the data, the standard approach in functional neuroimaging being feature selection (e.g.,* Anova*) [3, 6, 11, 12]. However, by performing feature selection and parameter estimation separately, such approach is not optimal. Thus, a popular combined selection/estimation scheme, *Recursive Feature Elimination* [13], may be used. However, this approach relies on a specific heuristic, which does not guarantee the optimality of the solution and is particularly costly. By contrast, there is great interest in sparsity-inducing regularizations, which optimize both simultaneously. 

In this paper, we assume that the code under investigation is about some scalar parameter that characterizes the stimuli, such as a scale/shape parameters but possibly also position, speed (assuming a 1-D space), or cardinality. Thus, we focus on regression problems and defer the generalization to classification to future work. Let us introduce the following predictive linear model:

(1) y=Xw+b,

where *y* represents the behavioral variable and (**w**, *b*) are the parameters to be estimated on a training set. A vector **w** ∈ ℝ^*p*^ can be seen as an image; *p* is the number of features (or voxels), and *b* ∈ ℝ is called the *intercept*. The matrix **X** ∈ ℝ^*n*×*p*^ is the design matrix. Each row is a *p*-dimensional sample, that is, an activation map related to the observation. With *n* ≪ *p*, the estimation of **w** is ill posed.

To cope with the high dimensionality of the data, one can penalize the estimation of **w**, for example, based on the *ℓ*_2_ norm of the weights. Classical regularization schemes have been used in functional neuroimaging, such as the Ridge regression [14], Lasso [15], or Elastic Net regression [16]. However, these approaches require the amount of penalization to be fixed beforehand and possibly optimized by cross-validation. To deal with the choice of the amount of penalization, one can use the Bayesian regression techniques, which include the estimation of regularization parameters in the whole estimation procedure. Standard Bayesian regularization schemes are based on the fact that a penalization by weighted *ℓ*_2_ norm is equivalent to setting the Gaussian priors on the weights **w**:

(2) $$\begin{array}{cc} & w\sim 𝒩\left(0,A^{-1}\right),\;A=\mathrm{diag}\;\left(\alpha_{1},\ldots ,\alpha_{p}\right),\;\\ & \;\;\;\;\;\forall i\in \left[1,\ldots ,p\right],\;\alpha_{i}\in {\mathbb{R}}^{+}, \end{array}$$

where *𝒩* is the Gaussian distribution and *α*_*i*_ the precision of the *i*th feature. The model in (2) defines two classical Bayesian regression schemes. The first one is *Bayesian Ridge Regression* (*BRR*) [17], which corresponds to the particular case *α*_1_ = ⋯ = *α*_*m*_. By regularizing all the features identically, BRR is not well suited when only few features are relevant. The second classical scheme is *Automatic Relevance Determination* (*ARD*) [18], which corresponds to the case *α*_*i*_ ≠ *α*_*j*_ if *i* ≠ *j*. The regularization performed by ARD is very adaptive, as all the weights are regularized differently. However, by regularizing each feature separately, ARD is prone to underfitting when the model contains too many regressors [19] and also suffers from convergence issues [20]. 

These classical Bayesian regularization schemes have been used in fMRI inverse inference studies [10, 14, 21]. However, these studies used only sparsity as built-in feature selection and do not consider neuroscientific assumptions for improving the regularization (i.e., within the design of the matrix *A*). Indeed, due to the intrinsic smoothness of functional neuroimaging data [22], predictive information is rather encoded in different groups of features sharing similar information. A potentially more adapted approach is the Bayesian regression scheme presented in [23], which regularizes patterns of voxels differently. The weights of the model are defined by **w** = *Uη*, where *U* is a matrix defined as set of spatial patterns (one pattern by column) and *η* are the parameters of the decomposition of **w** in the basis defined by *U*. The regularization is controlled through the covariance of *η*, which is assumed to be diagonal with only *m* possible different values cov (*η*) = exp  (*λ*_1_)**I**^(1)^ + ⋯+exp  (*λ*_*m*_)**I**^(**m**)^.

The matrices **I**^(**i**)^ are diagonal and defined subsets of columns of *U* sharing similar variance exp (*λ*_*i*_). Due to its class-based model, this approach is similar to the one proposed in this paper, but the construction of *I* relies on ad hoc voxel selection steps, so that there is no proof that the solution is correct. A contrario, the proposed approach jointly optimizes, within the same framework, the construction of the pattern of voxels and the regularization parameter of each pattern. 

In this paper, we detail a model for the Bayesian regression in which features are grouped into *K* different classes that are subject to different regularization penalties. The estimation of the penalty is performed in each class separately, leading to a stable and adaptive regularization. The construction of the group of features and the estimation of the predictive function are performed jointly. This approach, called *Multiclass Sparse Bayesian Regression (MCBR)*, is thus an intermediate solution between BRR and ARD. It requires less parameters to estimate than ARD and is far more adaptive than BRR. Another asset of the proposed approach in fMRI inverse inference is that it creates a clustering of the features and thus yields useful maps for brain mapping. After introducing our model and giving some details on the parameter estimation algorithms (the variational Bayes or Gibbs sampling procedures), we show that the proposed algorithm yields better accuracy than reference methods, while providing more interpretable models.

## 2. Multiclass Sparse Bayesian Regression

We first detail the notations of the problem and describe the priors and parameters of the model. Then, we detail the two different algorithms used for model inference.

### 2.1. Model and Priors

We recall the linear model for regression:

(3) y=f(X,w,b)=Xw+b.

We denote by **y** ∈ ℝ^*n*^ the targets to be predicted and **X** ∈ ℝ^*n*×*p*^ the set of activation images related to the presentation of different stimuli. The integer *p* is the number of voxels and *n* the number of samples (images). Typically, *p* ~ 10^3^  to  10^5^ (for a whole volume), while *n* ~ 10  to  10^2^.

**Priors on the Noise**

We use classical priors for regression, and we model the noise on **y** as an *i.i.d.* Gaussian variable:

(4) $$\begin{array}{c} \epsilon \sim 𝒩\left(0,\alpha^{-1}I_{n}\right),\\ \alpha \sim \Gamma \left(\alpha ;\alpha_{1},\alpha_{2}\right), \end{array}$$

where *α* is the precision parameter and Γ stands for the *gamma density* with two hyperparameters *α*_1_, *α*_2_:

(5) $$\Gamma \left(x;\alpha_{1},\alpha_{2}\right)=\alpha_{2}^{\alpha_{1}}x^{\alpha_{1}-1}\frac{{\exp\;}^{-x\alpha_{2}}}{\Gamma \left(\alpha_{1}\right)}.$$

**Priors on the Class Assignment**

In order to combine the sparsity of *ARD* with the stability of *BRR*, we introduce an intermediate representation, in which each feature *j* belongs to one class among *K* indexed by a discrete variable *z*_*j*_ (**z** = {*z*_1_,…, *z*_*p*_}).All the features within a class *k* ∈ {1,…, *K*} share the same precision parameter *λ*_*k*_, and we use the following prior on **z**:

(6) $$z\sim \overset{p}{\underset{j=1}{\prod }}\;\overset{K}{\underset{k=1}{\prod }}\pi_{k}^{\delta_{jk}},$$

where *δ* is *Kronecker's δ*, defined as

(7) $$\begin{array}{c} \delta_{jk}=\{\begin{array}{cc} 0 & \text{if}\;z_{j}\neq k,\\ 1 & \text{if}\;z_{j}=k. \end{array} \end{array}$$

We finally introduce an additional Dirichlet prior [24] on *π*:

(8) π~Dir(η)

with a hyperparameter *η*. By updating at each step the probability *π*_*k*_ of each class, it is possible to prune classes. This model has no spatial constraints and thus is not spatially regularized.

**Priors on the Weights**

**Figure 1:**
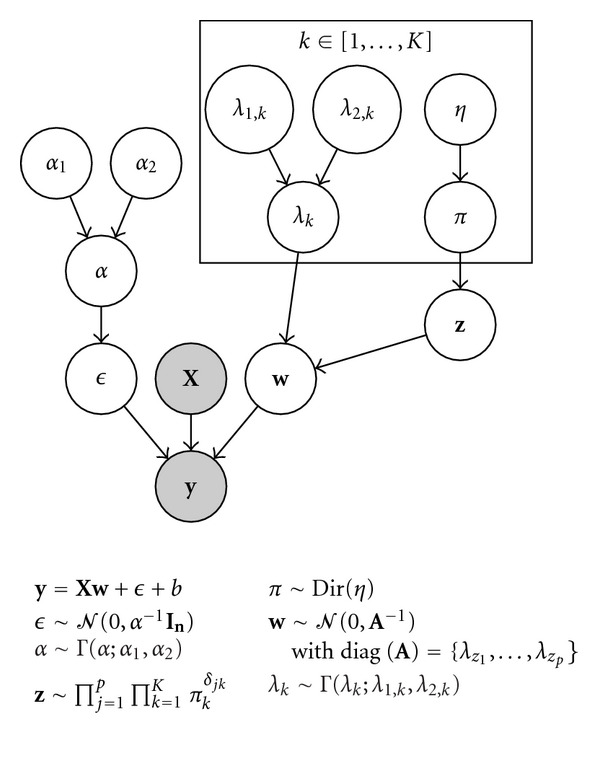
Graphical model of *Multiclass Sparse Bayesian Regression (MCBR)*. We denote by **y** ∈ ℝ^*n*^ the targets to be predicted and by **X** ∈ ℝ^*n*×*p*^ the set of activation images. both the weights of the model **w** depend on a discrete variable **z** that assigns each feature to a class *k* among *K*. Both the noise *ϵ* and the weights **w** have a Gamma prior on their precisions. The variable **z** follows a Dirichlet prior *π*.

As in ARD, we make use of an independent Gaussian prior for the weights:

(9) $$w\sim 𝒩\left(0,A^{-1}\right)\;\;\;\text{with}\;\mathrm{diag}\;\left(A\right)=\left\{\lambda_{z_{1}},\ldots ,\lambda_{z_{p}}\right\},$$

where *λ*_*z*_*j*__ is the precision parameter of the *j*th feature, with *z*_*j*_ ∈ {1,…, *K*}. We introduce the following prior on *λ*_*k*_:

(10) $$\begin{array}{c} \lambda_{k}\sim \Gamma \left(\lambda_{k};\lambda_{1,k},\lambda_{2,k}\right) \end{array}$$

with hyperparameters *λ*_1,*k*_, *λ*_2,*k*_. The complete generative model is summarized in Figure 1.

#### 2.1.1. Link with Other Bayesian Regularization Schemes

The link between the proposed MCBR model and the other regularization methods, Bayesian Ridge Regression and Automatic Relevance Determination, is obvious.

- With *K* = 1, that is, *λ*_*z*_1__ = ⋯ = *λ*_*z*_*p*__, we retrieve the BRR model,
- With *K* = *p*, that is, *λ*_*z*_*i*__ ≠ *λ*_*z*_*j*__ if *i* ≠ *j*, and assigning each feature to a singleton class (i.e., *z*_*j*_ = *j*), we retrieve the ARD model.

Moreover, the proposed approach is related to the one developed in [25]. In this paper, the authors proposed, for the distribution of weights of the features, a binary mixture of Gaussians with small and large precisions. This model is used for variable selection and estimated by the *Gibbs sampling*. Our work can be viewed as a generalization of this model to a number of classes *K* ≥ 2.

### 2.2. Model Inference

For models with latent variables, such as MCBR, some singularities can exist. For instance in a mixture of components, a singularity is a component with one single sample and thus zero variance. In such cases, maximizing the *log likelihood* yields flawed solutions, and one can use the posterior distribution of the latent variables *p*(**z** | **X**, **y**) for this maximization. However, the posterior distribution of the latent variables given the data does not have a closed-form expression, and some specific estimation methods, such as v*ariational Bayes* or *Gibbs sampling*, have to be used.

We propose two different algorithms for inferring the parameters of the MCBR model. We first estimate the model by the variational Bayes, and the resulting algorithm is thus called *VB-MCBR*. We also detail an algorithm, called *Gibbs-MCBR*, based on a Gibbs sampling procedure. 

#### 2.2.1. Estimation by Variational Bayes: VB-MCBR

The *variational Bayes* (or *VB*) approach provides an approximation *q*(Θ) of *p*(Θ | **y**), where *q*(Θ) is taken in a given family of distributions and Θ = [**w**, *λ*, *α*, **z**, *π*]. Additionally, the variational Bayes approach often uses the following *mean field approximation*, which allows the factorization between the approximate distribution of the latent variables and the approximate distributions of the parameters:

(11) $$\begin{array}{c} q\left(\Theta \right)=q\left(w\right)q\left(\lambda \right)q\left(\alpha \right)q\left(z\right)q\left(\pi \right). \end{array}$$

We introduce the *Kullback-Leibler* divergence *𝒟*(*q*(Θ)) that measures the similarity between the true posterior *p*(Θ | **y**) and the variational approximation *q*(Θ). One can decompose the *marginal log-likelihood *log *p* (**y**) as

(12) $$\begin{array}{c} \log\;p\;\left(y\;|\;\Theta \right)=ℱ\left(q\left(\Theta \right)\right)+𝒟\left(q\left(\Theta \right)\right) \end{array}$$

with

(13) $$\begin{array}{c} \begin{array}{c} ℱ\left(q\left(\Theta \right)\right)= \end{array}\int d\Theta q\left(\Theta \right)\log\;\;\frac{p\left(y,\Theta \right)}{q\left(\Theta \right)},\\ \begin{array}{c} 𝒟\left(q\left(\Theta \right)\right)= \end{array}\;\int d\Theta q\left(\Theta \right)\log\;\;\frac{q\left(\Theta \right)}{p\left(\Theta \;|\;y\right)}, \end{array}$$

where *ℱ*(*q*(Θ)) is called *free energy* and can be seen as the measure of the quality of the model. As *𝒟*(*q*(Θ)) ≥ 0, the free energy is a lower bound on log *p* (**y**) with equality if and only if *q*(Θ) = *p*(Θ | **y**). So, inferring the density *q*(Θ) of the parameters corresponds to maximizing *ℱ* with respect to the free distribution *q*(Θ). In practice, the *VB* approach consists in maximizing the free energy *ℱ* iteratively with respect to the approximate distribution *q*(**z**) of the latent variables and with respect to the approximate distributions of the parameters of the model *q*(**w**), *q*(*λ*), *q*(*α*), and *q*(*π*).

The variational distributions and the pseudocode of the VB-MCBR algorithm are provided in Appendix A. This algorithm maximizes the free energy *ℱ*. In practice, iterations are performed until convergence to a local maximum of *ℱ*. With an ARD prior (i.e., *K* = *p* and fixing *z*_*j*_ = *j*), we retrieve the same formulas as the ones found for *Variational ARD* [18].

#### 2.2.2. Estimation by Gibbs Sampling: Gibbs-MCBR

We develop here an estimation of the MCBR model using Gibbs sampling [26]. The resulting algorithm is called *Gibbs-MCBR*; the pseudocode of the algorithm and the candidate distributions are provided in Appendix B. The Gibbs sampling algorithm is used for generating a sequence of samples from the joint distribution to approximate marginal distributions. The main idea is to use conditional distributions that should be known and possibly easy to sample from, instead of directly computing the marginals from the joint law by integration (the joint law may not be known or hard to sample from). The sampling is done iteratively among the different parameters, and the final estimation of parameters is obtained by averaging the values of the different parameters across the different iterations (one may not consider the first iterations, this is called the *burn in*).

#### 2.2.3. Initialization and Priors on the Model Parameters

Our model needs few hyperparameters; we choose here to use slightly informative and class-specific hyperparameters in order to reflect a wide range of possible behaviors for the weight distribution. This choice of priors is equivalent to setting heavy-tailed centered *Student's t*-distributions with variance at different scales, as priors on the weight parameters. We set *K* = 9, with weakly informative priors *λ*_1,*k*_ = 10^*k*−4^, *k* ∈ [1,…, *K*] and *λ*_2,*k*_ = 10^−2^, *k* ∈ [1,…, *K*]. Moreover, we set *α*_1_ = *α*_2_ = 1. Starting with a given number of classes and letting the model automatically prune the classes can be seen as a means of avoiding costly model selection procedures. The choice of class-specific priors is also useful to avoid label switching issues and thus speeds up convergence. Crucially, the priors used here can be used in any regression problem, provided that the target data is approximately scaled to the range of values used in our experiments. In that sense, the present choice of priors can be considered as *universal*. We also randomly initialize *q*(**z**) for VB-MCBR (or **z** for Gibbs-MCBR). 

### 2.3. Validation and Model Evaluation

#### 2.3.1. Performance Evaluation

Our method is evaluated with a cross-validation procedure that splits the available data into training and validation sets. In the following, (**X**^*l*^, **y**^*l*^) are a learning set (**X**^*t*^, **y**^*t*^) is a test set, and ${\hat{y}}^{t}=F(X^{t}\hat{w})$ refers to the predicted target, where $\hat{w}$ is estimated from the training set. The performance of the different models is evaluated using *ζ*, the ratio of explained variance:

(14) $$\begin{array}{c} \zeta \left(y^{t},{\hat{y}}^{t}\right)=\frac{\text{var}\left(y^{t}\right)-\text{var}\left(y^{t}-{\hat{y}}^{t}\right)}{\text{var}\left(y^{t}\right)} \end{array}.$$

This is the amount of variability in the response that can be explained by the model (perfect prediction yields *ζ* = 1, while *ζ* < 0 if prediction is worse than chance).

#### 2.3.2. Competing Methods

 In our experiments, the proposed algorithms are compared to different state-of-the-art regularization methods.

- *Elastic Net Regression* [27], which requires setting two parameters *λ*_1_ and *λ*_2_. In our analyzes, a cross-validation procedure within the training set is used to optimize these parameters. Here, we use $\lambda_{1}\in \{0.2\tilde{\lambda },0.1\tilde{\lambda },0.05\tilde{\lambda },0.01\tilde{\lambda }\}$, where $\tilde{\lambda }=||X^{T}y{||}_{\infty }$, and *λ*_2_ ∈ {0.1,0.5,1., 10., 100.}. Note that *λ*_1_ and *λ*_2_ parametrize heterogeneous norms.
- *Support Vector Regression* (*SVR*) with a linear kernel [28], which is the reference method in neuroimaging. The *C* parameter is optimized by cross-validation in the range of 10^−3^ to 10^1^ in multiplicative steps of 10.
- *Bayesian Ridge Regression (BRR)*, which is equivalent to MCBR with *K* = 1 and *λ*_1_ = *λ*_2_ = *α*_1_ = *α*_2_ = 10^−6^, that is, weakly informative priors.
- *Automatic Relevance Determination (ARD)*, which is equivalent to MCBR with *K* = *p* and *λ*_1_ = *λ*_2_ = *α*_1_ = *α*_2_ = 10^−6^, that is, weakly informative priors.

All these methods are used after an *Anova*-based feature selection as this maximizes their performance. Indeed, irrelevant features and redundant information can decrease the accuracy of a predictor [29]. The optimal number of voxels is selected within the range {50,100,250,500}, using a nested cross-validation within the training set. We do not directly select a threshold on *P* value or cluster size, but rather a predefined number of features. The estimation of the parameters of the learning function is also performed using a nested cross-validation within the training set, to ensure a correct validation and an unbiased comparison of the methods. All methods are developed in *C* and used in *Python*. The implementation of elastic net is based on *coordinate descent* [30], while SVR is based on LibSVM [31]. Methods are used from *Python* via the *Scikit-learn* open source package [32].

 For VB-MCBR and Gibbs-MCBR, in order to avoid a costly *internal cross-validation*, we select 500 voxels, and this selection is performed on the training set. The number of iterations used is fixed to 5000 (*burn in* of 4000 iterations) for Gibbs-MCBR and 500 for VB-MCBR. Preliminary results on both simulated and real data showed that these values are sufficient enough for an accurate inference of the model. As explained previously, we set *K* = 9, with weakly informative priors *λ*_1,*k*_ = 10^*k*−4^, *k* ∈ [1,…, *K*] and *λ*_2,*k*_ = 10^−2^, *k* ∈ [1,…, *K*]. Moreover, we set *α*_1_ = *α*_2_ = 1, and we randomly initialize *q*(**z**) for VB-MCBR (or **z** for Gibbs-MCBR).

## 3. Experiments and Results

### 3.1. Experiments on Simulated Data

 We now evaluate and illustrate MCBR on two different sets of simulated data.

#### 3.1.1. Details on Simulated Regression Data

 We first test MCBR on a simulated data set, designed for the study of ill-posed regression problem, that is, *n* ≪ *p*. Data are simulated as follows:

(15) $$\begin{array}{cc} X & \sim 𝒩\left(0,1\right)\;\;\;\text{with}\;\epsilon \sim 𝒩\left(0,1\right),\\ y & =2\left(X_{1}+X_{2}-X_{3}-X_{4}\right)+0.5\left(X_{5}+X_{6}-X_{7}-X_{8}\right)+\epsilon . \end{array}$$

We have *p* = 200 features, *n*^*l*^ = 50 images for the training set, and *n*^*t*^ = 50 images for the test set. We compare MCBR to the reference methods, but we do not use feature selection, as the number of features is not very high.

#### 3.1.2. Results on Simulated Regression Data

We average the results of 15 different trials, and the average explained variance is shown in Table 1. Gibbs-MCBR outperforms the other approaches, yielding higher prediction accuracy than the reference elastic net and ARD methods. The prediction accuracy is also more stable than the other methods. VB-MCBR falls into the local maximum of *ℱ* and does not yield an accurate prediction. BBR has a low prediction accuracy compared to other methods such as ARD. Indeed, it cannot finely adapt the weights of the relevant features, as these features are regularized similarly as the irrelevant ones. SVR has also low accuracy, due to the fact that we do not perform any feature selection. Thus, SVR suffers from the curse of dimensionality, unlike other methods such as ARD or elastic net, which performs feature selection and model estimation jointly.

**Figure 2:**
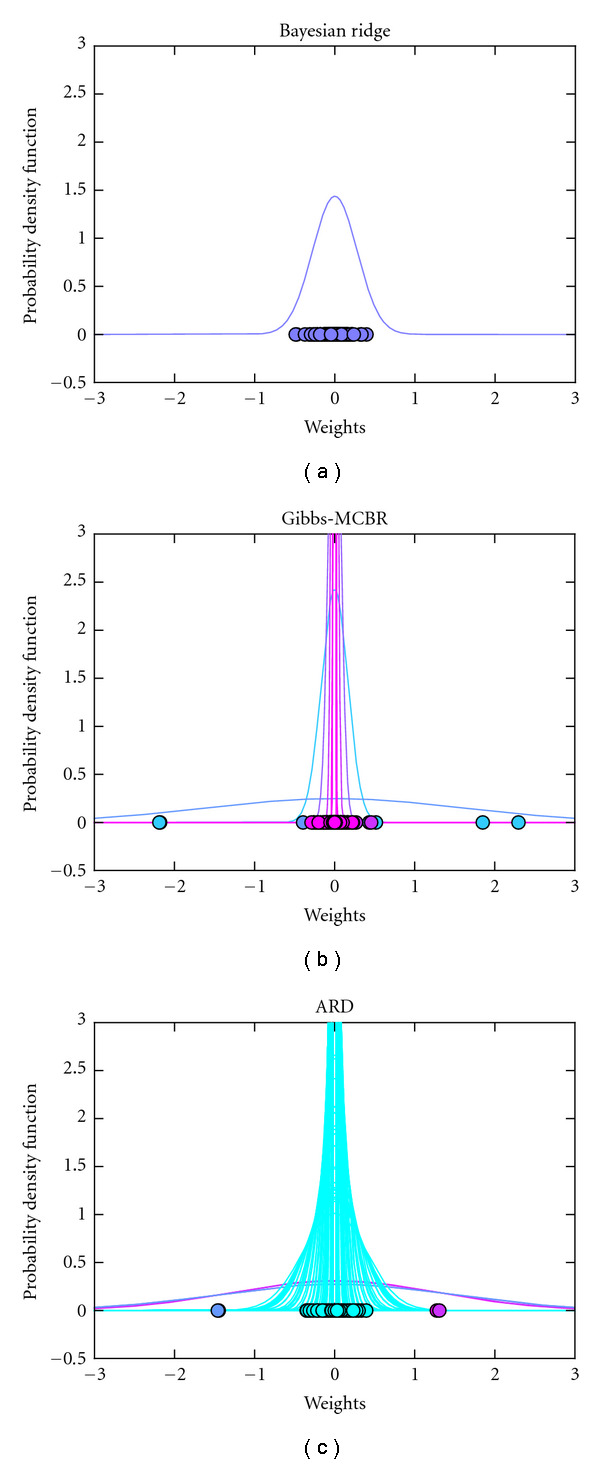
Results on simulated regression data. Probability density function of the weight distributions obtained with BRR (a), Gibbs-MCBR (b), and ARD (c). Each color represents a different component of the mixture model.

In Figure 2, we represent the probability density function of the distributions of the weights obtained with BRR (a), Gibbs-MCBR (b), and ARD (c). With BRR, the weights are grouped in a monomodal density. ARD is far more adaptive and sets lots of weights to zero. The Gibbs-MCBR algorithm creates a multimodal distribution, lots of weights being highly regularized (pink distributions), and informative features are allowed to have higher weights (blue distributions).

With MCBR, weights are clustered into different groups, depending on their predictive power, which is interesting in application such as fMRI inverse inference, as it can yield more interpretable models. Indeed, the class to the features with higher weights ({**X**_1_, **X**_2_, **X**_3_, **X**_4_}) belong which is small (average size of 6 features) but has a high *purity* (percentage of relevant features in the class) of 74%.

#### 3.1.3. Comparison between VB-MCBR and Gibbs-MCBR

**Figure 3:**
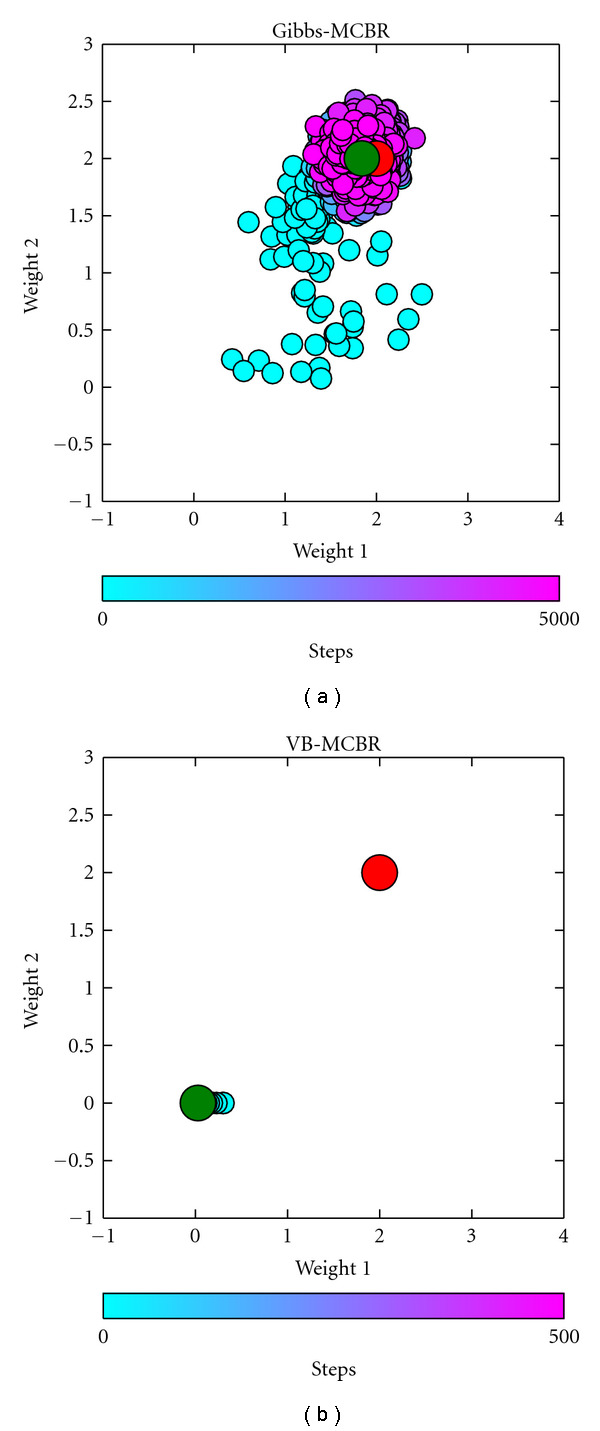
Results on simulated regression data. Weights of the first two features found for the different steps of Gibbs-MCBR (a) and VB-MCBR (b). The red dot represents the ground truth of both weights, and the green dot represents the final state found by the two algorithms. VB-MCBR is stuck in a local maximum, and Gibbs-MCBR finds the correct weights.

We now look at the values of *w*_1_ and *w*_2_ for the different steps of the two algorithms (see Figure 3). We can see that VB-MCBR (b) quickly falls into a local maximum, while Gibbs-MCBR (a) visits the space and reaches the region of the correct set of parameters (red dot). VB-MCBR is not optimal in this case. 

### 3.2. Simulated Neuroimaging Data

#### 3.2.1. Details on Simulated Neuroimaging Data

The simulated data set **X** consists of *n* = 100 images (size 12 × 12 × 12 voxels) with a set of four square regions of interest (ROI) (size 2 × 2 × 2). We call *ℛ* the support of the ROI (i.e., the 32 resulting voxels of interest). Each of the four ROIs has a fixed weight in {−0.5,0.5, −0.5,0.5}. We call *w*_*i*,*j*,*k*_ the weight of the (*i*, *j*, *k*) voxel. The resulting images are smoothed with a Gaussian kernel with a standard deviation of 2 voxels, to mimic the correlation structure observed in real fMRI data. To simulate the spatial variability between images (intersubject variability, movement artifacts in intrasubject variability), we define a new support of the ROIs, called $\tilde{ℛ}$ such that, for each image *l*th, 50% (randomly chosen) of the weights **w** are set to zero. Thus, we have $\tilde{ℛ}\subset ℛ$. We simulate the target **y** for the *l*th image as

(16) $$\begin{array}{c} y_{l}=\underset{(i,j,k)\in \tilde{ℛ}}{\sum }w_{i,j,k}X_{i,j,k,l}+\epsilon_{l} \end{array}$$

with the signal in the (*i*, *j*, *k*) voxel of the *l*th image simulated as

(17) $$\begin{array}{c} X_{i,j,k,l}\sim 𝒩\left(0,1\right) \end{array},$$

and *ϵ*_*l*_ ~ *𝒩*(0, *γ*) is a Gaussian noise with standard deviation *γ* > 0. We choose *γ* in order to have a signal-to-noise ratio of 5 dB.

#### 3.2.2. Results on Simulated Neuroimaging Data

**Figure 4:**
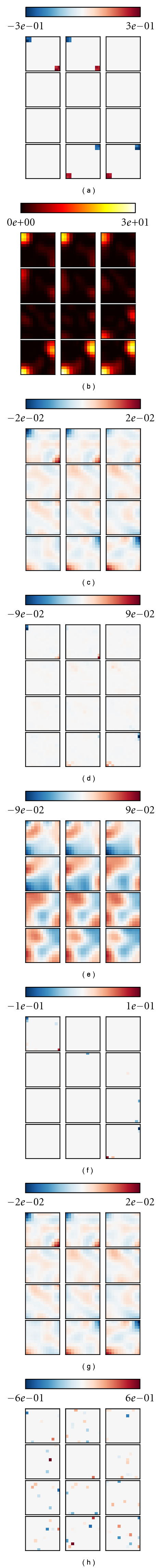
Two-dimensional slices of the three-dimensional volume of simulated data. Weights found by different methods, the true target (a) and F-score (b). The Gibbs-MCBR method (d) retrieves almost the whole spatial support for the weights. The sparsity-promoting reference methods, elastic net (f) and ARD (h), find an overly sparse support of the weights. VB-MCBR (c) converges to a local maximum similar to BRR (g) and thus does not yield a sparse solution. SVR (e) yields smooth maps that are not similar to the ground truth.

We compare VB-MCBR and Gibbs-MCBR with the different competing algorithms. The resulting images of weights are given in Figure 4, with the true weights (a) and resulting Anova F-scores (b). The reference methods can detect the truly informative regions (*ROIs*), but elastic net (f) and ARD (h) retrieve only part of the support of the weights. Moreover, elastic net yields an overly sparse solution. BRR (g) also retrieves the *ROIs* but does not yield a sparse solution, as all the features are regularized in the same way. We note that the weights in the *feature space* estimated by SVR (e) are nonzero everywhere and do not outline the support of the ground truth. VB-MCBR (c) converges to a local maximum similar to the solution found by BRR (g); that is, it creates only one nonempty class, and thus regularizes all the features similarly. We can thus clearly see that, in this model, the variational Bayes approach is very sensitive to the initialization and can fall into nonoptimal local maxima, for very sparse support of the weights. Finally, Gibbs-MCBR (d) retrieves most of the true support of the weights by performing an adapted regularization.

### 3.3. Experiments and Results on Real fMRI Data

In this section, we assess the performance of MCBR in an experiment on the *mental representation of object size*, where the aim is to predict the size of an object seen by the subject during the experiment, in both intrasubject and intersubject cases. The size (or scale parameter) of the object will be the target variable **y**.

#### 3.3.1. Details on Real Data

We apply the different methods on a real fMRI dataset related to an experiment studying the representation of objects, on ten subjects, as detailed in [33]. During this experiment, ten healthy volunteers viewed objects of 4 shapes in 3 different sizes (yielding 12 different experimental conditions), with 4 repetitions of each stimulus in each of the 6 sessions. We pooled data from the 4 repetitions, resulting in a total of *n* = 72 images by subject (one image of each stimulus by session). Functional images were acquired on a 3-T MR system with an eight-channel head coil (Siemens Trio, Erlangen, Germany) as T2*-weighted echo-planar image (EPI) volumes. Twenty transverse slices were obtained with a repetition time of 2 s (echo time: 30 ms; flip angle: 70°; 2 × 2 × 2-mm voxels; 0.5 mm gap). Realignment, normalization to MNI space, and general linear model (GLM) fit were performed with the SPM5 software (http://www.fil.ion.ucl.ac.uk/spm/software/spm5/). The normalization is the conventional method of SPM (implying affine and nonlinear transformations) and not the one using unified segmentation. The normalization parameters are estimated on the basis of a whole-head EPI acquired in addition and are then applied to the partial EPI volumes. The data are not smoothed. In the GLM, the effect of each of the 12 stimuli convolved with a standard hemodynamic response function was modeled separately, while accounting for serial autocorrelation with an AR(1) model and removing low-frequency drift terms using a high-pass filter with a cutoff of 128 s. The GLM is fitted separately in each session for each subject, and we used in the present work the resulting session-wise parameter estimate images (the *β*-maps are used as rows of **X**). The four different shapes of objects were pooled across for each one of the three sizes, and we are interested in finding discriminative information on sizes. This reduces to a regression problem, in which our goal is to predict a simple scalar factor (size of an object). All the analyzes are performed without any prior selection of regions of interest and use the whole acquired volume.

**Intrasubject Regression Analysis**

First, we perform an intrasubject regression analysis. Each subject is evaluated independently, in a 12-fold cross-validation. The dimensions of the real data set for one subject are *p* ~ 7 × 10^4^ and *n* = 72 (divided in 3 different sizes, 24 images per size). We evaluate the performance of the method by a leave-one-condition-out cross-validation (i.e., leave-6-image-out), and doing so the GLM is performed separately for the training and test sets. The parameters of the reference methods are optimized with a nested leave-one-condition-out cross-validation within the training set, in the ranges given before.

**Intersubject Regression Analysis**

Additionally, we perform an intersubject regression analysis on the sizes. The intersubject analysis relies on subject-specific fixed-effect activations that is, for each condition, the 6 activation maps corresponding to the 6 sessions are averaged together. This yields a total of 12 images per subject, one for each experimental condition. The dimensions of the real data set are *p* ~ 7 × 10^4^ and *n* = 120 (divided into 3 different sizes). We evaluate the performance of the method by cross-validation (leave-one-subject-out). The parameters of the reference methods are optimized with a nested leave-one-subject-out cross-validation within the training set, in the ranges given before.

#### 3.3.2. Results on Real Data

**Intrasubject Regression Analysis**

The results obtained by the different methods are given in Table 2. The *P-values* are computed using a paired *t*-test across subjects. VB-MCBR outperforms the other methods. Compared to the results on simulated data, VB-MCBR still falls in a local maximum similar to the Bayesian ridge regression which performs well in this experiment. Moreover, both Gibbs-MCBR and VB-MCBR are more stable than the reference methods.

**Intersubject Regression Analysis**

The results obtained with the different methods are given in Table 3. As in the intrasubject analysis, both MCBR approaches outperform the reference methods, SVR, BRR, and ARD. However, the prediction accuracy is similar to that of elastic net. In this case, Gibbs-MCBR performs slightly better than VB-MCBR, but the difference is not significant.

**Figure 5:**
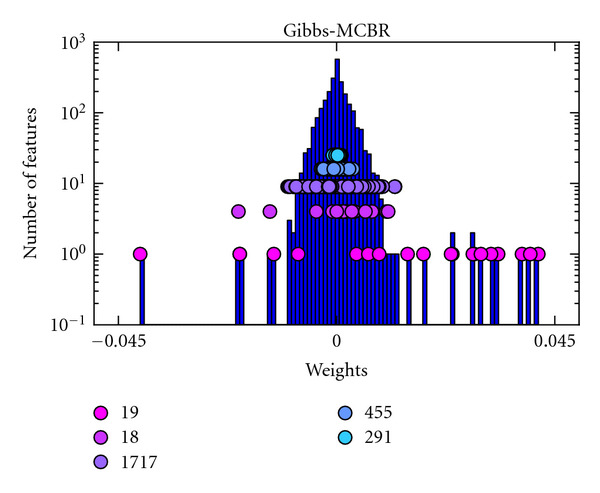
*Intersubject analysis*. Histogram of the weights found by Gibbs-MCBR and corresponding **z** values (each color of dots represents a different class), for the intersubject analyzes. We can see that Gibbs-MCBR creates clusters of informative and noninformative voxels and that the different classes are regularized differently, according to the relevance of the features in each of them.

One major asset of MCBR (and more particularly Gibbs-MCBR, as VB-MCBR often falls into a one-class local maximum) is that it creates a clustering of the features, based on the relevance of the features in the predictive model. This clustering can be accessed using the variable **z**, which is implied in the regularization performed on the different features. In Figure 5, we give the histogram of the weights of Gibbs-MCBR for the intersubject analysis. We keep the weights and the values of **z** of the last iteration; the different classes are represented as dots of different colors and are superimposed on the histogram. We can notice than the pink distribution represented at the bottom of the histogram corresponds to relevant features. This cluster is very small (19 voxels), compared to the two blue classes represented at the top of the histogram that contain many voxels (746 voxels) which are highly regularized, as they are noninformative.

**Figure 6:**
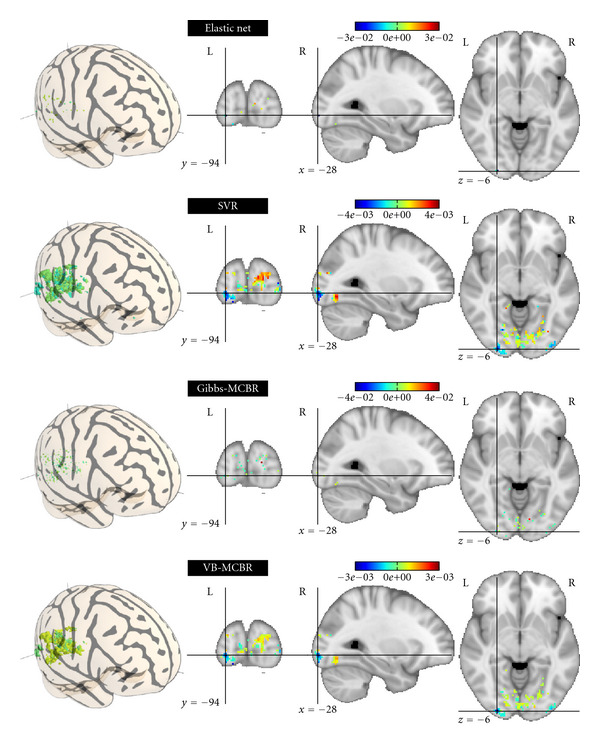
*Intersubject analysis*. Maps of weights found by the different methods on the 2500 most relevant features by Anova. The map found by elastic net is difficult to interpret as the very few relevant features are scattered within the whole brain. SVR and VB-MCBR do not yield a sparse solution. Gibbs-MCBR, by performing an adaptive regularization, draws a compromise between the other approaches and yields a sparse solution, but also extracts small groups of relevant features.

The maps of weights found by the different methods are detailed in Figure 6. The methods are used combined with an Anova-based *univariate feature selection* (2500 voxels selected, in order to have a good support of the weights). As elastic net, Gibbs-MCBR yields a sparse solution but extracts a few more voxels. The map found by elastic net is not easy to interpret, with very few informative voxels scattered in the whole occipital cortex. The map found by SVR is not sparse in the *feature space* and is thus difficult to interpret, as the spatial layout of the neural code is not clearly extracted. VB-MCBR does not yield a sparse map either, all the features having nonnull weights

## 4. Discussion

It is well known that in high-dimensional problems, regularization of feature loadings significantly increases the generalization ability of the predictive model. However, this regularization has to be adapted to each particular dataset. In place of costly cross-validation procedures, we cast regularization in a Bayesian framework and treat the regularization weights as hyperparameters. The proposed approach yields an adaptive and efficient regularization and can be seen as a compromise between a global regularization (Bayesian Ridge Regression) that does not take into account the sparse or focal distribution of the information and automatic relevance determination. Additionally, MCBR creates a clustering of the features based on their relevance and thus explicitly extracts groups of informative features.

Moreover, MCBR can cope with the different issues of ARD. ARD is subject to an underfitting in the hyperparameter space that corresponds to an underfitting in model selection (i.e., on the features to be pruned) [19]. Indeed, as ARD is estimated by maximizing evidence, models with less selected features are preferred, as the integration is done on less dimensions, and thus evidence is higher. ARD will choose the sparsest model across models with similar accuracy. A contrario, MCBR requires far less hyperparameter (2 × *K*, with *K* ≪ *p*) and suffers less from this issue, as the sparsity of the model is defined by groups. Moreover, a full Bayesian framework for estimating ARD requires to set some priors on the *hyperparameters* (e.g., *α*_1_ and *α*_2_), and it may be sensitive to specific choice of these hyperparameters. A solution is to use an *internal cross-validation* for optimizing these parameters, but this approach can be computationally expensive. In the case of MCBR, the distributions of the hyperparameters are bound to a class and not to each feature. Thus, the proposed approach is less sensitive to the choice of the hyperparameters. Indeed, the choice of good hyperparameters for the features is dealt with at the class level.

On simulated data, our approach performs better than other classical methods such as SVR, BRR, ARD, and elastic net and yields a more stable prediction accuracy. Moreover, by adapting the regularization to different groups of voxels, MCBR retrieves the true support of the weights and recovers a sparse solution. Results on real data show that MCBR yields more accurate predictions than other regularization methods. As it yields less sparse solution than elastic net, it gives access to more plausible loading maps which are necessary for understanding the spatial organization of brain activity, that is, retrieving the spatial layout of the neural coding. On real fMRI data, the explicit clustering of Gibbs-MCBR is also an interesting aspect of the model, as it can extract few groups of relevant features from many voxels.

In some experiments, the variational Bayes algorithm yields less accurate predictions than the Gibbs sampling approach, which can be explained by the difficulty of initializing the different variables (especially **z**) when the support of the weight is overly sparse. Moreover, the VB-MCBR algorithm relies on a variational Bayes approach, which may not be optimal, due to strong approximations in model inference. A contrario Gibbs-MCBR is more time consuming but yields a better model inference. Finally, the variability in the results may be explained by the difficulty to estimate the model (optimality is not ensured).

The question of model selection (i.e., the number of classes *K*) has not been addressed in this paper. One can use the free energy in order to select the best model, but due to the instability of VB-MCBR, this approach does not seem promising. A more interesting method is the one detailed in [34], which can be used with the Gibbs sampling algorithm. Here, model selection is performed implicitly by emptying classes that do not fit the data well. In that respect, the choice of heterogeneous priors for different classes is crucial: replacing our priors with class-independent priors (i.e., *λ*_1,*k*_ = 10^−2^, *k* ∈ [1,…, *K*]) in the intersubject analysis on size prediction leads Gibbs-MCBR to a local maximum similar to VB-MCBR.

Finally, this model is not restricted to the Bayesian regularization and can be used for classification, within a probit or logit model [35, 36]. The proposed model may thus be used for diagnosis in medical imaging, for the prediction of both continuous or discrete variables.

## 5. Conclusion

In this paper, we have proposed a model for adaptive regression, called *MCBR*. The proposed method integrates, in the same Bayesian framework, BRR and ARD and performs a different regularization for relevant and irrelevant features. It can tune the regularization to the possible different level of sparsity encountered in fMRI data analysis, and it yields interpretable information for fMRI inverse inference, namely, the **z** variable (latent class variable). Experiments on both simulated and real data show that our approach is well suited for neuroimaging, as it yields accurate and stable predictions compared to the state-of-the-art methods.

## Figures and Tables

**Algorithm 1:**
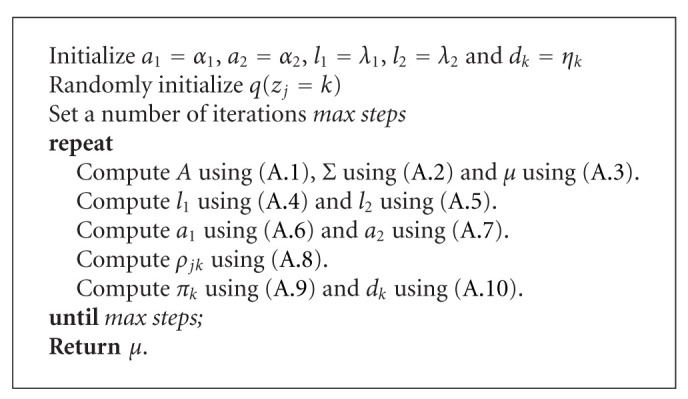
VB-MCBR algorithm.

**Algorithm 2:**
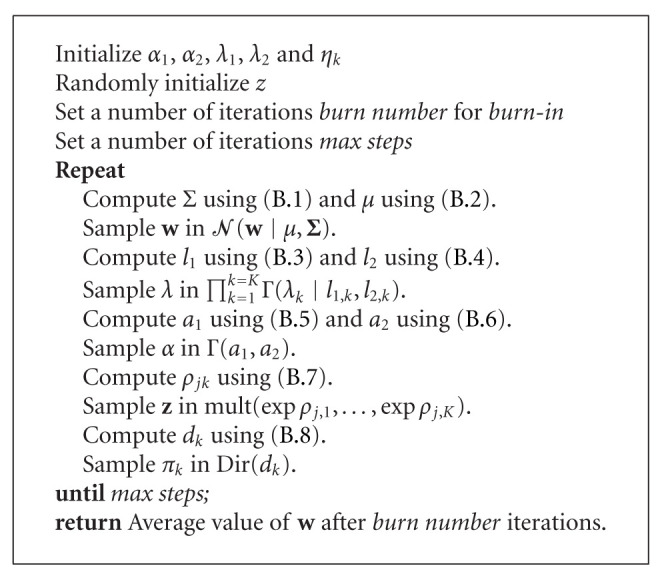
Gibbs-MCBR algorithm.

**Table 1:** *Simulated regression data*. Explained variance *ζ* for different methods (average of 15 different trials). The *P-*values are computed using a paired *t*-test.

Methods	Mean *ζ*	Std *ζ*	*P*-value to Gibbs-MCBR
SVR	0.11	0.1	.0**
Elastic net	0.77	0.11	.0004**
BRR	0.19	0.14	.0**
ARD	0.79	0.06	.0**
Gibbs-MCBR	0.89	0.04	—
VB-MCBR	0.04	0.05	.0**

**Level of significance of the *P*-values between 0.01 and 0.05.

**Table 2:** *Intrasubject analysis*. Explained variance *ζ* for the three different methods. The *P*-values are computed using a paired *t*-test. VB-MCBR yields the best prediction accuracy, while being more stable than the reference methods.

Methods	Mean *ζ*	Std *ζ*	*P*-val/Gibbs-MCBR
SVR	0.82	0.07	.0006***
Elastic net	0.9	0.02	.001***
BRR	0.92	0.02	.0358**
ARD	0.89	0.03	.0015***
Gibbs-MCBR	0.93	0.01	—
VB-MCBR	0.94	0.01	.99

**Level of significance of the *P*-values between 0.01 and 0.05.

***Level of significance of the *P*-values below 0.01.

**Table 3:** *Intersubject analysis*. Explained variance *ζ* for the different methods. The *P*-values are computed using a paired *t*-test. MCBR yields highest prediction accuracy than the two other Bayesian regularizations BRR and ARD.

Methods	Mean *ζ*	Std *ζ*	*P*-val/Gibbs-MCBR
SVR	0.77	0.11	.14
Elastic net	0.78	0.1	.75
BRR	0.72	0.1	.01**
ARD	0.52	0.33	.02*
Gibbs-MCBR	0.79	0.1	—
VB-MCBR	0.78	0.1	0.4

*Level of significance of the *P*-values.

**Level of significance of the *P*-values between 0.01 and 0.05.

## Appendices

### A. VB-MCBR Algorithm

The *variational Bayes* approach yields the following variational distributions:

(i) *q*(**w**) ~ *𝒩*(**w** | *μ*, *𝚺*) with
  (A.1) $$\begin{array}{cc} \bar{A} & =\mathrm{diag}\;\left(\bar{l_{1}},\ldots ,\bar{l_{p}}\right)\;\text{with}\\ \bar{l_{j}} & =\overset{K}{\underset{k=1}{\sum }}q\left(z_{j}=k\right)\frac{l_{1,k}}{l_{2,k}}\;\;\forall j\in \left\{1,\ldots ,p\right\}, \end{array}$$
  (A.2) $$\begin{array}{cc} \text{Σ} & ={\left(\frac{a_{1}}{a_{2}}X^{T}X+\bar{A}\right)}^{-1}, \end{array}$$
  (A.3) $$\begin{array}{cc} \mu & =\frac{a_{1}}{a_{2}}\text{Σ}X^{T}y \end{array};$$
(ii) *q*(*λ*_*k*_) ~ Γ(*l*_1,*k*_, *l*_2,*k*_) with
  (A.4) $$\begin{array}{c} \begin{array}{c} l_{1,k}=\lambda_{1,k}+\frac{1}{2}\overset{p}{\underset{j=1}{\sum }}q\left(z_{j}=k\right), \end{array} \end{array}$$
  (A.5) $$\begin{array}{c} \begin{array}{c} l_{2,k}=\lambda_{2,k}+\frac{1}{2}\overset{p}{\underset{j=1}{\sum }}\left(\mu_{jj}^{2}+\Sigma_{jj}\right)q\left(z_{j}=k\right); \end{array} \end{array}$$
(iii) *q*(*α*) ~ Γ(*a*_1_, *a*_2_) with
  (A.6) $$\begin{array}{c} a_{1}=\alpha_{1}+\frac{n}{2}, \end{array}$$
  (A.7) $$\begin{array}{c} a_{2}=\alpha_{2}+\frac{1}{2}{\left(y-X\mu \right)}^{T}\left(y-X\mu \right)+\frac{1}{2}\mathrm{Tr}\;\left(\text{Σ}X^{T}X\right) \end{array};$$
(iv) *q*(*z*_*j*_ = *k*) ~ exp ^(*ρ*_*jk*_)^ with
  (A.8) $$\begin{array}{c} \rho_{jk}=-\frac{1}{2}\left(\mu_{j}^{2}+\Sigma_{jj}\right)\frac{l_{1,k}}{l_{2,k}}+\ln\;\left(\pi_{k}\right)+\frac{1}{2}\left(\Psi \left(l_{1,k}\right)-\log\;\left(l_{2,k}\right)\right) \end{array},$$
  (A.9) $$\begin{array}{c} \pi_{k}={\exp\;}^{\left\{\Psi (d_{k})-\Psi (\sum_{k=1}^{k=K}d_{k})\right\}}, \end{array}$$
  (A.10) $$\begin{array}{c} d_{k}=\eta_{k}+\overset{p}{\underset{j=1}{\sum }}q\left(z_{j}=k\right), \end{array}$$
  where Ψ is the digamma function Ψ(*x*) = Γ′(*x*)/Γ(*x*). The *VB-MCBR* algorithm is provided in pseudo-code in Algorithm 1.

### B. Gibbs-MCBR Algorithm

With Θ = [**w**, *λ*, *α*, **z**, *π*], we have the following candidate distributions (i.e., the distributions used for the sampling of the different parameters):

(i) *p*(**w** | Θ − {**w**}) ∝ *𝒩*(**w** | *μ*, *𝚺*) with
  (B.1) $$\begin{array}{c} \text{Σ}={\left(X^{T}X\alpha +A\right)}^{-1}\;\;\;\;\text{with}\;A=\mathrm{diag}\;\left(\lambda_{z_{1}},\ldots ,\lambda_{z_{p}}\right) \end{array},$$
  (B.2) $$\begin{array}{c} \mu =\text{Σ}\alpha X^{T}y \end{array};$$
(ii) *p*(*λ* | Θ − {*λ*}) ∝ ∏_*k*=1_^*K*^Γ(*λ*_*k*_ | *l*_1,*k*_, *l*_2,*k*_) with
  (B.3) $$\begin{array}{c} l_{1,k}=\lambda_{1,k}+\frac{1}{2}\overset{p}{\underset{j=1}{\sum }}\delta \left(z_{j}=k\right), \end{array}$$
  (B.4) $$\begin{array}{c} l_{2,k}=\lambda_{2,k}+\frac{1}{2}\overset{p}{\underset{j=1}{\sum }}\delta \left(z_{j}=k\right)w_{j}^{2} \end{array};$$
(iii) *p*(*α* | Θ − {*α*}) ∝ Γ(*a*_1_, *a*_2_) with
  (B.5) $$\begin{array}{c} a_{1}=\alpha_{1}+\frac{n}{2}, \end{array}$$
  (B.6) $$\begin{array}{c} a_{2}=\alpha_{2}+\frac{1}{2}{\left(y-X\mu \right)}^{T}\left(y-X\mu \right) \end{array};$$
(iv) *p*(*z*_*j*_ | Θ − {**z**}) ∝ mult(exp *ρ*_*j*,1_,…, exp *ρ*_*j*,*K*_) with
  (B.7) $$\begin{array}{c} \rho_{jk}=-\frac{1}{2}w_{j}^{2}\lambda_{k}+\ln\;\;\left(\pi_{k}\right)+\frac{1}{2}\log\;\;\lambda_{k} \end{array};$$
(v) *p*(*π*_*k*_ | Θ − {*π*}) ∝ Dir(*d*_*k*_) with
  (B.8) $$\begin{array}{c} d_{k}=\eta_{k}+\overset{p}{\underset{j=1}{\sum }}\delta \left(z_{j}=k\right). \end{array}$$

The algorithm is provided in pseudocode in Algorithm 2.
